# Proper Food for Children

**Published:** 1892-09

**Authors:** Mary F. Russell


HALL'S
JOURNAL OF HEALTH

TRUTH DEMANDS NO SACRIFICE; ERROR CAN MAKE NONE.

Vol. 39. SEPTEMBER, 1892. No. 9.

### PROPER FOOD FOR CHILDREN.

The instincts of healthy children should certainly be permitted to have weight as regards their diet, and this is perhaps especially true as to the quantity of food they require. A healthy, well-born child is probably a better judge of the amount of food it requires than its parent. We do not arbitrarily stop a baby nursing when it is eagerly devouring its meal, for we have learned that when it has become satisfied it will cease to eat of its own accord.

Instinct must certainly be relied upon, but we must remember that children are not ideally healthy, and that because they and we are so far from nature we must have recourse to science for help in this as in other subjects.

As regards quantity, then, we must have in mind that a child requires relatively more food than his elders, for obvious reasons. The parent has certain brain and muscular work to accomplish, the power for which comes mainly from his food. He must also maintain his bodily heat, and this he is constantly expending in work as well as in radiating from his body. This heat and these losses must be made good by food. But the child of the school age, from 7 to 14 years, must not only do all this but much more. He must not only hold his own, but must daily add new material to his little body, and lay up a plus balance to meet the demands of his rapidly growing organism.

Children suffer more during famine than adults, and it is probable that with them more danger is incurred by underfeeding than by overfeeding. They certainly require an abundance.

Of what shall this abundance consist? What shall the growing child eat?

If we were to analyze the constitution of our food in general, we should find that we eat nitrogen and carbon and hydrogen and phosphorus in various forms, as well as iron and soda and potash with other minerals in proportions that vary with the kind of diet we adopt. And we have very good reason for believing that some of these food stuffs contribute more directly than others to bodily energy, and that this energy may be increased or diminished by giving or withholding certain of these elements.

When, for instance, a man is training for a boat race—an effort calling for muscular exertion, as well as nervous endurance—he lives largely upon meat, a nitrogen-supplying food. When he has to endure great cold he seeks fatty food—carbo-hydrate food, as evidenced in the Eskimos and other dwellers in Arctic regions.

What are the physical requirements of the growing child? He is developing bone and muscle at an enormous rate, and all of his internal organs must keep pace with this general growth.

His height, for instance, which means the growth of his skeleton, increases on an average from 1¾ inches to 2 inches per year up to the age of 12, and for the succeeding two or three years (longer for a boy) he may increase in some cases from 3 to 4 inches. His weight gains 4 to 10 pounds yearly, in some cases more than this. He is making brain, too, as well as using up nervous energy all of this time.

Now, while all the food-stuffs that we eat have a general relation to growth and nutrition, certain elements have a peculiar adaptability to building up special tissues. Lean meat offers one of the most digestible and concentrated forms of obtaining nitrogen, which it contains principally in the form of albumen, and nitrogenous food especially promotes muscular as well as nervous energy, and the energy obtained from this food appears to be liberated more quickly than from other stuffs, It is also believed that such food has special influence in forming red blood cells. Albumen (nitrogenous food) is not, however, by any means confined to animal food. Certain grains, unbolted wheat, and oats contain a large amount of nitrogen, as do also some of the leguminous foods, such as peas and beans and lentils.

Milk contains nitrogen in the form of albumen and casein. So that both as a digestible and a highly nutritious food it should be included in the dietary of the growing child.

But besides energy we must furnish heat, and while this, it is true, is gained by the interworking of all the organs, and by the chemical activities constantly going on within us, so that all kinds of nourishment may help to furnish the heat required, yet it is true that fat is the food that is most useful in this respect.

The heat forming qualities of fat are nearly double those of lean meat, for while a pound of lean meat, if oxygenated, will yield heat enough to raise over 4,000 pounds of water one degree centigrade, the same quantity of fat will raise over 9,000 pounds of water to the same point. So we can see how much more economical in cold climates is an admixture of fat with lean meat for heating purposes merely. But fat is useful for other reasons, and we judge it to be a very necessary article of diet from its forming so large a proportion of some of the most important tissues of the body—namely, the nervous—the brain containing 8 per cent. of fat while the nerves contain 22 per cent. It must not be understood that fat in the tissues is made directly from fat in the food : on the contrary more fat is continually being manufactured in the body than can be accounted for in the food. One use for fat is believed to be as a protector of the albumen, which is less rapidly used if fat be taken with it, and practically we see every day the value of administering fat to children of our climate and national temperament, and the harm that may result from withholding it.

A distinguished English physician once said that the two great causes of tuberculosis were the dearness of butter and the abundance of pastry cooks. It is a well known fact that children are frequently chided for eating too much butter on their bread, when the real reason of the denial is not its unwholesomeness, but its expense, and when their own instinct for the food which they need is every day shown to be in that respect a natural and healthy one.

It is well proven that in scrofulous affections, as well as in pulmonary tuberculosis, the use of fat in the form of cream. olive oil, butter or cod liver oil is attended with the best of results, and forms, indeed, an important prescription with every experienced practitioner. These fats are also commonly given in nervous exhaustion with the happiest results.

These and similar facts certainly indicate that this form of a carbohydrate element may well be used to prevent as well as to cure these constitutional tendencies. I believe, in fact, that many of our thin and pale children who have tendencies to colds and sore throats, and many others who are irritable, would be greatly benefited by the continued and judicious use of fat in some digestible form, such as fat bacon, the use of cream on bread or baked potatoes, as well as by a bountiful supply of good butter. All of these fats will be more digestible if well subdivided, hence we spread butter on our bread and may use bread crumbs or potatoes with fat bacon. Chocolate or cocoa is also useful for a fat provider.

But these highly nutritious articles must be mixed with less concentrated food or the digestive organs will be overtaxed, and so we must give the children starch, which is another form of carbon. Rice, potatoes and refined flour are good examples of this food stuff. The proportion that starchy food should bear to albumen and fatty foods is much greater in the diet of an adult than in that of a child. An adult takes about three times as much starchy food as of the other two combined, but a child requires more of the albumen and fat, proportionately, and therefore somewhat less starch than the above.

Too many children receive an excess of starchy and too little albuminous and fatty food, and this diet tends to induce amæmia, to form bone poorly and to reduce nerve strength.

How many children, for instance, are allowed to make an entire breakfast upon griddle cakes made of fine wheat flour, which is so refined as to have lost the larger part of its nutritive elements except starch, with molasses added. The molasses in this case only adds more carbon, like the starch, and here is an entire meal furnished to a little active body which contains hardly a vestige of nitrogen, or muscle-makers, and very little of other most important nerve foods, and on this poor meal the child must study the whole morning. Not infrequentlv he is too tired on returning home at luncheon time to eat the chop or steak provided and perhaps satisfies his appetite with baked apples and gingerbread, the latter being made, as was the morning meal, from "highly refined" wheat flour, and therefore mainly a starchy food. Such food will not make brawn and brain for our children.

It is well known as regards flour, for instance, that certain of its highly nutritive elements are more or less lost by the process of refinement to which they are put, and hence bread and other articles made from flour do not afford the nourishment which many mothers suppose themselves to be offering in such food.

In the wheat the nitrates (muscle-makers) as well as the phosphates, lie mainly in the outer layers of the little grain, the inner kernel being largely composed of starch. So large a proportion of these elements is lost by refining that it is estimated that bran contains fourteen times as much of the phosphates and nitrates as ordinary superfine flour. Gluten is also wasted in this process, so that it would seem to be a loss both to the organism of the child and to the household economy as well.—*Dr. Mary F. Russell.*

